# Supplementary figures and images for: Factors Predictive of Primary Resistance to Immune Checkpoint Inhibitors in Patients with Advanced Non-Small Cell Lung Cancer
Source: Cancers (Basel). 2023 May 12;15(10):2733. doi: 10.3390/cancers15102733 (PMC10216169; doi:10.3390/cancers15102733)

# Overall survival

Strata — No Primary Resistance — Primary Resistance

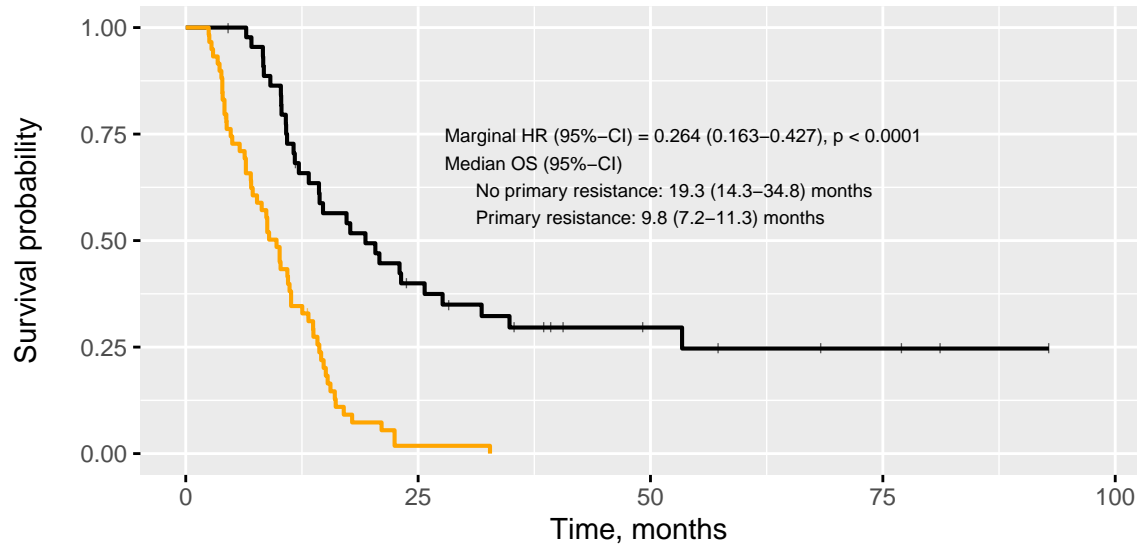

## Number at risk

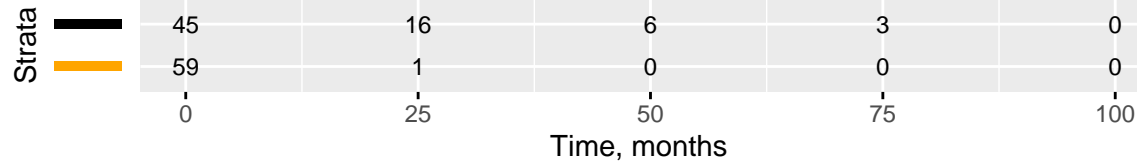

Supplement: Supplementary file 1 [file cancers-15-02733-s001.zip › cancers-2345465-supplementary.pdf]
